# Monocytes and macrophages in malignant melanoma IV. Effects of C. parvum on monocyte function.

**DOI:** 10.1038/bjc.1979.101

**Published:** 1979-05

**Authors:** D. W. Hedley, R. E. Nyholm, G. A. Currie

## Abstract

Assays for the capacity of peripheral-blood monocytes (a) to mature in vitro into macrophages, (b) to reduce nitro-blue tetrazolium (NBT) and (c) to lyse antibody-coated human Group A red cells, were applied to a group of 82 patients with histologically proven malignant melanoma. In patients with micrometastatic disease there was an enhancement of red-cell lysis and NBT reduction, suggesting that their monocytes are in some way "activated", whereas NBT reduction was suppressed in those with overt dissemination. Monocyte maturation in vitro was impaired in all patient groups to an extent which correlated with overall tumour burden. Corynebacterium parvum was administered i.v. to 12 patients with disseminated disease and by the intradermal route to 24 patients with micrometastatic disease. The 3 monocyte functions were significantly enhanced by C. parvum.


*Br. J. Cancer* (1979) **39**, 558

### MONOCYTES AND MACROPHAGES IN MALIGNANT MELANOMA IV. EFFECTS OF *C. PARVUM* ON MONOCYTE FUNCTION

D. W. HEDLEY, R. E. NYHOLM AND G. A. CURRIE

*From the Division of Tumour Immunology, Chester Beatty Research Institute, and The Royal Marsden Hospital, Belmont, Sutton, Surrey*

Received 12 December 1978 Accepted 12 January 1979

**Summary.—Assays for the capacity of peripheral-blood monocytes (a) to mature *in vitro* into macrophages, (b) to reduce nitro-blue tetrazolium (NBT) and (c) to lyse antibody-coated human Group A red cells, were applied to a group of 82 patients with histologically proven malignant melanoma. In patients with micrometastatic disease there was an enhancement of red-cell lysis and NBT reduction, suggesting that their monocytes are in some way "activated", whereas NBT reduction was suppressed in those with overt dissemination. Monocyte maturation *in vitro* was impaired in all patient groups to an extent which correlated with overall tumour burden.**

***Corynebacterium parvum* was administered i.v. to 12 patients with disseminated disease and by the intradermal route to 24 patients with micrometastatic disease. The 3 monocyte functions were significantly enhanced by *C. parvum*.**

THERE IS INCREASING EVIDENCE from animal studies that the mononuclear phagocyte system (MPS) may play an important effector role in host responses to malignant tumours (Alexander, 1976) and that the administration of *Corynebacterium parvum* can exert anti-tumour activity by inducing proliferation and activation of cells in the MPS (Scott, 1974). Monocyte function in patients with malignant melanoma is disturbed. Using assays designed to examine 3 different aspects of monocyte function (differentiation, NBT reduction and lysis of opsonized erythrocytes) we have previously obtained evidence (Currie & Hedley, 1977; Hedley & Currie, 1978) that the detectable abnormalities may be related to clinical tumour burden. In brief, these earlier studies showed that, in patients with minimal tumour burden, peripheral blood monocytes, although showing defective differentiation, are "activated" relative to age-matched control donors, and that these monocyte functions become depressed in patients with overt disseminated disease. We now report a more detailed examination of a larger series of patients which firmly establishes correlations between clinical tumour burden and certain monocyte functions. We have also examined the effects of administration of *C. parvum* to selected subgroups of patients to determine whether this agent can enhance the functions of peripheral-blood monocytes, and to examine the effect of its route, dosage and timing of administration, in order to design an optimally effective, minimally toxic regimen for subsequent clinical evaluation.

### MATERIALS AND METHODS

*Patients*

A total of 82 patients attending the melanoma clinic at this hospital were investigated. Blood samples were taken before the administration of any systemic treatment. No patient was investigated within 3 weeks of prior surgery.

These patients as a group were a highly selected population in that they all had received prior surgery. Forty-five patients had clinically overt disseminated disease. The remaining 37 were those with an undetectable tumour burden following surgery, but with a poor prognosis. They comprised patients with deeply penetrating primary tumours (Clark's Levels IV and V), surgically treated Stage IIB disease or surgically treated recurrent local or distant cutaneous disease. We refer to the disease in these patients as "micrometastatic". An unknown but small number were probably cured by the surgery and did not have micrometastatic disease, but since we are unable to identify them they cannot be excluded from these investigations. Details of the groups of patients receiving *C. parvum* will be given below. Normal control blood samples were obtained from a group of 48 heterogeneous normal donors. They were not rigorously age-matched since we have been unable to detect any effect of age on monocyte function in panels of normal donors.

### *Assays of monocyte function*

Mononuclear-cell suspensions were prepared from defibrinated peripheral venous blood using the method of Boyum (1968), and examined by enzyme cytochemistry (Yam *et al.*, 1971). The percentage of cells staining diffusely for the monocyte marker non-specific esterase (NSE) was estimated and, because of the possibility of errors in the monocyte-function assays due to contaminating granulocytes, the latter cells were also counted using chloroacetate-esterase activity as a marker.

*Monocyte maturation.*—In brief, this method examines the ability of monocytes to mature into macrophages when cultured in 50% fresh autologous serum at 37°C for 7 days in the wells of 3040 (Falcon Plastics) microplates (Currie & Hedley, 1977). The macrophage nuclei were detached and stained using a solution of 0·1M citric acid plus 1 : 2000 crystal violet, and counted in a haemacytometer. Results were expressed as the percentage of $NSE^+$ cells maturing into macrophages.

*Monocyte NBT reduction.*—The capacity of monocytes to reduce the dye nitro-blue tetrazolium was measured by a quantitative assay (Hedley & Currie, 1978). The rate of dye reduction is an indirect measure of hexose-monophosphate-shunt activity, the enhancement of which is associated with monocyte "activation".

Mononuclear cells were pre-incubated for 15 min in the presence of latex polystyrene particles, which acted as a phagocytic stimulus, after which a solution of NBT was added.

After 60 min incubation with NBT the reaction was stopped by acidifying the mixture, the cells were washed and then reduced, insoluble NBT was extracted into dioxan at 70°C. Optical density at 520 nm was measured and, by reference to a standardization curve, the amount of NBT reduced per $NSE^+$ cell, both resting and after latex preincubation, could be calculated.

*Lysis of antibody-coated enthrocytes.*—The ability of peripheral-blood monocyte to lyse antibody-coated human erythrocytes was measured using the method of Nyholm & Currie (1978). Fresh human Group A red cells were labelled with sodium ($^{51}$Cr)chromate, trypsinized and coated with a hyperimmune anti-$A_1$ serum. Fifty microlitres of Medium 199 containing $10^5$ red cells were added to the wells of 3040 plates (Falcon Plastics) containing serial dilutions of mononuclear cells ranging from 1·0 to $5{\cdot}0 \times 10^5$ in 50 $\mu$l. The plates were incubated at 37°C for 2 h and the release of $^{51}$Cr counted in an automatic gamma counter. The results were then expressed as a percentage of $^{51}$Cr release determined as follows:

$$\%\ ^{51}\mathrm{Cr\ release} = \frac{\text{Release in test well} \atop \text{—spontaneous release}}{\text{Total releasable} \atop \text{—spontaneous release}} \times 100$$

The total releasable $^{51}$Cr was measured after the addition of 5% sodium dodecyl sulphate.

A parameter was then derived describing the number of red cells lysed per $NSE^+$ cell.

### *Administration of C. parvum*

*C. parvum* (Coparvax, The Wellcome Foundation Limited), was administered to 2 groups of patients. A group of 12 patients with overt disseminated disease was given *C. parvum* by the i.v. route. A range of starting doses from 700 $\mu$g to 7 mg were infused i.v. diluted in 200 ml dextrose-saline over a period of 30 min. The patients were closely observed for the next 48 h. Blood samples for routine haematological and biochemical investigations were taken at frequent intervals, and monocyte-function tests performed before infusion and at intervals thereafter. No other medication was given unless indicated by symptoms.

A further series of 24 patients with "micrometastatic" disease were given *C. parvum* by the intradermal (i.d.) route. The vaccine was diluted in sterile physiological saline to a final concentration of 700 $\mu$g/ml and was given by i.d. injection in 0·1ml volumes into the deltoid region (of an arm uninvolved by tumour) in doses ranging from 70 $\mu$g to 560 $\mu$g at intervals (2, 3, or 4 weeks) for a total period of 6 months. These 24 patients were a heterogeneous group, including 16 with surgically treated Stage IIB disease, 3 with level IV or V primary tumours, 3 with recurrent primary tumours, 1 rectal primary and 1 with a single distant cutaneous metastasis.

### RESULTS

### *Monocyte function and tumour burden*

*Monocyte maturation*.—Data obtained from 82 patients and 30 normal donors expressed as percent maturation, are shown in Table I and Fig. 1. The patients, after conventional clinical staging, were divided into those with "micrometastatic" disease and those with overt disseminated disease. The patients in the latter category were further subdivided into those whose disease was apparently confined to skin, lymph nodes and/or lungs and those where disease involved liver, brain, bone or viscera. As can be seen from the data there are statistically significant differences between each of the groups (*i.e.* monocyte maturation in normal donors>micrometastatic disease>disseminated disease). Furthermore, in the latter group, those with disease in skin lymph node and/or lung show significantly higher values than those with visceral, brain or bone disease.

*Quantitative NBT reduction*.—The data obtained in 48 normal donors and 60 patients with malignant melanoma are seen in Table I. In 34 patients with micrometastatic disease the resting NBT reduction was $12{\cdot}4 \pm 5{\cdot}3$ (s.d.)$\times 10^{-15}$ mol/$NSE^+$ cell, whereas in the normal donors the value obtained was $10{\cdot}4 \times 3{\cdot}3$. This difference became more apparent after latex stimulation (normals $15{\cdot}2 \pm 4{\cdot}9 \times 10^{-15}$ mol/$NSE^+$ cell, micrometastatic disease $18{\cdot}0 \pm 6{\cdot}3$; $P < 0{\cdot}05$). Furthermore, the patients with overt disseminated disease showed significantly depressed NBT reduction ($8{\cdot}9 \pm 4{\cdot}1 \times 10^{-15}$ mol/$NSE^+$ cell) which responded poorly to stimulation by latex particles ($11{\cdot}6 \pm 3{\cdot}9 \times 10^{-15}$ mol/$NSE^+$ cell). Again, patients with visceral metastases showed the most severe defect.

*Lysis of antibody-coated erythrocytes*.—The results of this assay were used to derive a parameter describing the number of antibody-coated Group A red cells

TABLE I.—*Correlations of clinical tumour stage with results from 3 assays of monocyte function*

| | Normal (n) | | Melanomas: Micro-metastatic (n) | | All disseminated (n) | | Skin, node, or lung metastases (n) | | Visceral metastases (n) |
|---|---|---|---|---|---|---|---|---|---|
| | (30) | | (37) | | (45) | | (18) | | (27) |
| Maturation % | $47{\cdot}1 \pm 22{\cdot}3$ | | $28{\cdot}2 \pm 16{\cdot}0$ | | $14{\cdot}8 \pm 12{\cdot}1$ | | $20{\cdot}9 \pm 14{\cdot}5$ | | $10{\cdot}7 \pm 8{\cdot}9$ |
| | | $P<0{\cdot}001$ | | $P<0{\cdot}001$ | | *1 | | $P<0{\cdot}02$ | |
| Resting NBT: | (48) | | (34) | | (26) | | (13) | | (13) |
| Reduction fmol/monocyte | $10{\cdot}4 \pm 3{\cdot}3$ | | $12{\cdot}4 \pm 5{\cdot}3$ | | $8{\cdot}9 \pm 4{\cdot}1$ | | $10{\cdot}2 \pm 4{\cdot}4$ | | $7{\cdot}5 \pm 3{\cdot}3$ |
| | | N.S. | | $P<0{\cdot}01$ | | *2 | | N.S. | |
| Stimulated NBT: | (48) | | (34) | | (26) | | (13) | | (13) |
| Reduction fmol/monocyte | $15{\cdot}2 \pm 4{\cdot}9$ | | $18{\cdot}0 \pm 6{\cdot}3$ | | $11{\cdot}9 \pm 3{\cdot}9$ | | $13{\cdot}4 \pm 3{\cdot}6$ | | $9{\cdot}9 \pm 3{\cdot}3$ |
| | | $P<0{\cdot}05$ | | $P<0{\cdot}001$ | | *3 | | $P<0{\cdot}02$ | |
| Red cells | (39) | | (32) | | (23) | | (11) | | (12) |
| lysed/monocyte | $0{\cdot}56 \pm 0{\cdot}35$ | | $0{\cdot}92 \pm 5{\cdot}1$ | | $0{\cdot}77 \pm 0{\cdot}42$ | | $0{\cdot}81 \pm 0{\cdot}51$ | | $0{\cdot}73 \pm 0{\cdot}32$ |
| | | $P<0{\cdot}001$ | | N.S. | | *4 | | N.S. | |

*P* values are derived from data in adjacent columns (except * when comparison is normal v. all disseminated).
*1. $P<0{\cdot}001$.
*2. N.S.
*3. $P<0{\cdot}001$.
*4. $P<0{\cdot}05$.

FIG. 1.—Percentage of monocytes maturing into macrophages according to disease stage.

lysed per monocyte (defined as NSE$^+$ cells). The assay was performed on 39 normal donors who provided a mean value of $0{\cdot}56 \pm 0{\cdot}35$ red cells/monocyte, whereas 32 patients with micrometastatic disease gave a mean of $0{\cdot}92 \pm 0{\cdot}51$ red cells/monocyte, a difference which is highly significant ($P < 0{\cdot}001$) and indicates that monocytes from such patients show increased lytic activity. In a smaller group of 23 patients with disseminated disease the mean value was $0{\cdot}77 \pm 0{\cdot}42$ red cells/monocyte, a figure significantly higher than that from the normal donors ($P < 0{\cdot}05$) (Table I).

*Effects of C. parvum*

*Hazards of i.v. administration.*—When given by this route in doses ranging from 700 μg to 7 mg the toxic side effects comprised fever, chills, nausea, rigors, headache, tachycardia, transient hypertension or hypotension: a syndrome described by the patients as like severe influenza, and which persisted for 2–3 days. One patient collapsed and died 16 h after an i.v. infusion of *C. parvum*, and we therefore did not feel justified in continuing to use this agent by the i.v. route. This patient, a man aged 41 with widespread pulmonary metastases, was treated according to a protocol comprising alternating *C. parvum* and chemotherapy (DTIC and vincristine) at 2-week intervals. About 16 h after his second i.v. infusion of 7 mg *C. parvum* he collapsed and died. Subsequent post-mortem examination showed no evidence of cardiovascular disease or intravascular coagulation, and confirmed the presence of extensive lung deposits of malignant melanoma. The immediate cause of his death remains unexplained.

Since the i.v. route was abandoned on the grounds of toxicity, the data obtained from studies of monocyte-function were limited. However, results from the monocyte maturation assay performed before and 7–14 days after the first infusion of *C. parvum* showed substantial (and significant) increases in monocyte maturation which was not related to the dose of *C. parvum* (*i.e.* patients given 700 μg provided results similar to those receiving 7 mg; data not shown).

*Intradermal administration.*—When given by this route *C. parvum* was without toxicity. At the local injection site there were mild indurated erythematous reactions, resembling delayed-type hypersensitivity. These were never severe, and never led to ulceration, scarring or abscess formation. There was no symptomatic, haematological or biochemical evidence of systemic toxicity.

A total of 24 patients were given *C. parvum* by the i.d. route and *in vitro* data were obtained on 21. The monocyte-function assays were performed before the first injection and at intervals thereafter. The detailed results are shown in Table II, which describes the effects on monocyte function after the first i.d. injection of *C. parvum* in these 21 patients. In general, *C. parvum* i.d. caused a significant eleva-

TABLE II—*Monocyte function before and after a single intradermal injection of* C. parvum

| Melanoma Patient No. | Disease Stage | Dose (μg) | Interval (weeks) | Monocyte Maturation % | | NBT Reduction (fmol/Monocyte) Resting | | NBT Reduction (fmol/Monocyte) Stimulated | | RBC lysis/Monocyte | |
|---|---|---|---|---|---|---|---|---|---|---|---|
| | | | | Before | After | Before | After | Before | After | Before | After |
| 517 | IIB | 105 | 3 | 50·0 | 83·0 | 9·2 | 30·6 | 18·4 | 57·7 | 1·79 | 2·78 |
| 565 | IIB | 105 | 3 | 21·0 | 16·3 | 5·0 | 31·7 | 10·0 | 30·2 | ND | ND |
| 578 | IIB | 140 | 3 | 5·5 | 16·5 | 5·2 | 4·7 | ND | ND | ND | ND |
| 585 | Rectal Primary | 140 | 3 | 21·8 | 52·6 | 15·3 | 16·0 | 19·4 | 19·0 | ND | ND |
| 556 | Distant Cutaneous | 140 | 2 | 21·0 | 67·8 | 10·9 | 33·0 | 15·5 | 39·2 | 0·81 | 1·33 |
| 604 | Clark IV | 140 | 2 | 24·5 | 21·3 | 13·0 | 18·0 | 16·2 | 22·8 | 0·35 | 0·68 |
| 576 | IIB | 140 | 3 | 20·1 | 78·4 | 3·3 | 13·4 | ND | ND | ND | ND |
| 594 | IIB | 140 | 3 | 16·0 | 10·0 | 8·6 | 11·9 | 12·2 | 16·1 | ND | ND |
| 588 | IIB | 140 | 3 | 4·4 | 90·0 | 4·7 | 28·3 | 7·0 | 35·2 | ND | ND |
| 607 | Clark IV | 140 | 3 | 12·0 | 23·8 | 20·7 | 43·1 | 26·0 | 45·4 | 1·20 | 1·04 |
| 584 | Clark V | 140 | 4 | 33·1 | 27·2 | 8·5 | 9·3 | ND | ND | ND | ND |
| 601 | IIB | 140 | 4 | 50·8 | 10·5 | 13·8 | 7·6 | ND | ND | ND | ND |
| 569 | Recurrent Nasal | 210 | 4 | 5·0 | 22·8 | 13·9 | 28·9 | 16·6 | 36·5 | 0·70 | 1·43 |
| 611 | IIB | 280 | 2 | 11·0 | 34·2 | 9·8 | 25·6 | 14·4 | 25·6 | 0·71 | 0·68 |
| 624 | IIB | 280 | 2 | 36·4 | 57·4 | 12·3 | 25·9 | 24·1 | 25·9 | ND | ND |
| 625 | IIB | 280 | 2 | 30·6 | 13·5 | 13·6 | 14·7 | 16·2 | 17·1 | 0·69 | 0·97 |
| 620 | Recurrent Primary | 350 | 2 | 51·0 | 34·2 | 11·9 | 28·4 | 12·7 | 23·6 | 0·53 | 1·00 |
| 629 | IIB | 350 | 2 | 20·6 | 44·4 | 19·0 | 30·4 | 21·2 | 44·5 | 0·46 | 1·61 |
| 635 | Recurrent Primary | 350 | 3 | 13·3 | 126·6 | 15·1 | 27·7 | 15·7 | 33·6 | 0·29 | 0·81 |
| 640 | IIB | 560 | 2 | 42·1 | 25·3 | 17·9 | 6·0 | 21·0 | 7·7 | 0·78 | 0·71 |
| 636 | IIB | 560 | 3 | 23·4 | 16·7 | 5·5 | 8·2 | 8·6 | 9·0 | 0·38 | 1·47 |

tion in the functions assayed. Using the Wilcoxon test for matched pairs, the injection of *C. parvum* caused a significant rise in monocyte maturation ($P<0{\cdot}05$), a rise in unstimulated NBT reduction ($P<0{\cdot}01$) and a rise in stimulated NBT reduction ($P<0{\cdot}01$). The assay for lysis of opsonized erythrocytes also showed a significant increase ($P<0{\cdot}01$).

A range of doses from 105 μg to 560 μg revealed no clear dose relationship in effects of any of the assays. Continued i.d. injections of *C. parvum* (at 2-, 3- or 4-weekly intervals) led to complex patterns of rise and fall of the assays. In 4 patients (1 after the first injection, the other 3 after multiple injections) the monocyte-maturation assay provided values well over 100%. In other words, proliferation of cells was occurring *in vitro*. Mitotic figures were seen in these cultures, a phenomenon never seen in cultures from untreated patients or normal donors. We interpret this finding as evidence for a population of circulating progenitor cells evoked by the *C. parvum*.

Fig. 2.—Monocyte maturation in 6 patients with micrometastatic melanoma and receiving repeated intradermal *C. parvum* (as arrowed) : ●——● remaining disease-free; ○——○R, early relapse.

Fig. 3.—Monocyte NBT reduction ($10^{-15}$ fmol/NSE$^+$ cell) in patients with micrometastatic melanoma and receiving repeated intradermal *C. parvum* (as arrowed) ●——● remaining disease-free; ○——○R, early relapse.

Since the start of this study (16 months) 3 patients in this micrometastatic group receiving i.d. *C. parvum* have relapsed with metastatic disease. The results of their monocyte functions are shown in Figs 2 and 3, and are compared to those of 3 clinically similar patients who have not so far shown any evidence of recurrent disease. Monocyte maturation and NBT reduction in these 3 early-relapse patients show a small transient rise followed by a fall coincident with (or in 2 cases preceding) clinically detectable recurrence. The patients not relapsing showed a greater response to *C. parvum*, which was sustained for much longer (3–4 months). However, even in these cases (with no sign of relapse) receiving periodic *C. parvum* injections, the responses (both maturation and NBT reduction) eventually subsided towards normal levels after 3–4 months.

### DISCUSSION

### *Monocyte functions and clinical stage*

Since Dizon & Southam's (1963) demonstration of defective macrophage mobilization in malignant disease, many attempts have been made to examine cells of the MPS in such patients. A variety of functional abnormalities of peripheral-blood monocytes have been described, including increased hexose-monophosphate-shunt activity (King *et al.*, 1977), increased expression of Fc receptors (Lobuglio, 1970; Rhodes, 1977) and defective chemotaxis (Boetcher & Leonard, 1974). Our own studies have been concerned with three assays for various aspects of monocyte function, and include maturation *in vitro* into macrophages, quantitative reduction of nitro-blue tetrazolium (NBT) and the capacity to lyse antibody-coated human erythrocytes. The results indicate that the capacity of monocytes to mature *in vitro* clearly correlates with disease burden. Patients with disease disseminated to viscera, bone or brain, and therefore with the worst prognosis (Luce, 1972; Einhorn *et al.*, 1974), show the lowest % maturation. Those with micrometastatic disease (*i.e.* clinically disease-free but with a very high risk of recurrence) provide much higher values, which are still significantly less than the control donor values. There was no correlation between suppression of monocyte maturation and the presence of granulocytes or other contaminant cells in the mononuclear-cell suspensions. Preliminary results indicate that suppression of monocyte differentiation is mediated by labile factor(s) in the patient's sera. NBT reduction, however, shows a more complex pattern. Monocytes from patients with disseminated disease show depressed resting NBT reduction and a deficient capacity to respond to a phagocytic stimulus. However, patients with micrometastatic disease provided values for resting NBT reduction above the normal donor values, and after a phagocyte stimulus showed a hypernormal response. We interpret this as evidence for some form of as yet uncharacterized activation. This observation is supported by the earlier work of King *et al.* (1977) and by the data from the erythrocyte-lysis assay. Rhodes (1977) has reported the increased expression of Fc receptors on the monocytes from cancer patients, perhaps another aspect of "activation". These findings all suggest an important role for the MPS in host responses to a malignant tumour.

*Effects of C. parvum*

Manipulation of the MPS constitutes one of the major (and as yet unsuccessful) approaches to the immunotherapy of human cancer (Terry & Windhorst, 1978).

*Corynebacterium parvum* has been given to patients with a variety of diseases by various routes in a range of doses. The ability of *C. parvum* to modify functions of the MPS in man has been hinted at by studies of the clearance of $^{125}$I-labelled aggregated albumin (Attié, 1975) and the development of a monocytosis (Israel, 1975). Our own studies were performed in order to determine the optimal (and safest) mode of administering *C. parvum* (*i.e.* route, dose and timing). *C. parvum* injected i.v. produced unacceptably toxic side effects. When given i.d., however, it was without adverse effects and raised all 3 monocyte functions examined: maturation, NBT reduction and lytic capacity. There was no obvious influence of dose: 105 μg produced changes similar to those at much higher doses. Repeated administration at 2- or 3-week intervals led to a progressive rise in the monocyte functions in most of the patients. There was a suggestion from these data that 2- or 3-week intervals provide a better long-term stimulating effect on monocyte function than intervals of 4 weeks. In 4 patients given i.d. *C. parvum* there was evidence of *in vitro* proliferation of monocytes, as shown by the presence of mitotic figures in the cultures and eventual maturation indices in excess of 100%. This observation is reminiscent of the known effects of *C. parvum* on marrow colony-forming cells (Wolmark & Fisher, 1974). We have never encountered this phenomenon in the patients or normal donors not receiving *C. parvum*. Three of the patients showed only minor and transient responses to *C. parvum* followed by rapidly subsiding monocyte function. Each of these 3 relapsed early with overt disseminated disease, the collapse of monocyte functions preceding clinical detection of metastatic disease.

Since the i.d. administration of *C.*

*parvum* every 2–3 weeks in doses around 100–400 μg can produce a series of changes in monocyte function interpretable as stimulation and/or activation, such a protocol is being evaluated as an adjuvant form of therapy.

Studies in these laboratories are supported by a programme grant from the Medical Research Council. G.A.C. gratefully acknowledges support from the Cancer Research Campaign. We thank Dr T. J. McElwain for close collaboration and helpful discussions.

### REFERENCES

ALEXANDER, P. (1976) The functions of the macrophage in malignant disease. *Ann. Rev. Med.*, **27**, 207.

ATTIÉ, E. (1975) Action of *Corynebacterium parvum* on the phagocytic activity of the reticuloendothelial system in cancer patients. In Corynebacterium parvum. *Applications in Experimental and Clinical Oncology*. Ed. B. Halpern, New York: Plenum Press. p. 341.

BOETCHER, D. A. & LEONARD, E. J. (1974) Abnormal monocyte chemotactic response in cancer patients. *J. Natl Cancer Inst.*, **52**, 1091.

BÖYUM, A. (1968) Isolation of mononuclear cells and granulocytes from human blood. *Scand. J. Clin. Lab. Invest.*, **21**, 77.

CURRIE, G. A. & HEDLEY, D. W. (1977) Monocytes and macrophages in malignant melanoma. 1. Peripheral blood macrophage precursors. *Br. J. Cancer*, **36**, 1.

DIZON, Q. & SOUTHAM, C. M. (1963) Abnormal cellular responses to skin abrasions in cancer patients. *Cancer*, **16**, 1288.

EINHORN, I. H., BURGESS, M. A., VALLEJOS, C. & 9 others (1974) Prognostic correlations and response to treatment in advanced metastatic malignant melanoma. *Cancer Res.*, **34**, 1994.

HEDLEY, D. W. & CURRIE, G. A. (1978) Monocytes and macrophages in malignant melanoma III. Reduction of nitroblue tetrazolium by peripheral blood monocytes. *Br. J. Cancer*, **37**, 747.

ISRAEL, L. (1975) Report on 414 cases of human tumours treated with Corynebacteria. In Corynebacterium parvum. *Applications in Experimental and Clinical Oncology*. Ed. B. Halpern. New York: Plenum Press. p. 402.

KING, G. W., LOBUGLIO, A. F. & SAGONE, A. L. (1977) Human monocyte metabolism in lymphoma. *J. Lab. Clin. Med.*, **89**, 316.

LOBUGLIO, A. F. (1970) Effect of neoplasia on human macrophage activity. *J. Lab. Clin. Med.*, **76**, 888.

LUCE, J. (1972) Chemotherapy of malignant melanoma. *Cancer*, **30**, 1604.

NYHOLM, R. E. & CURRIE, G. A. (1978) Monocytes and macrophages in malignant melanoma II. Lysis of antibody-coated erythrocytes as an assay of monocyte function. *Br. J. Cancer*, **37**, 339.

RHODES, J. (1977) Altered expression of human monocyte Fc receptors in malignant disease. *Nature*, **265**, 253.

SCOTT, M. T. (1974) *Corynebacterium parvum* as a therapeutic anti-tumour agent in mice. 1. Systemic effects from intravenous injection. *J. Natl Cancer Inst.*, **53**, 855.

TERRY, W. D. & WINDHORST, D., Eds. (1978) *Immunotherapy of Cancer: Present status of trials in Man*. New York: Raven Press.

WOLMARK, N. & FISHER, B. (1974) The effect of a single and repeated administration of *Corynebacterium parvum* on bone marrow macrophage colony production in syngeneic tumour-bearing mice. *Cancer Res.*, **34**, 2869.

YAM, L. T., LI, C. Y. & CROSBY, W. H. (1971) Cytochemical identification of monocytes and granulocytes. *Am. J. Clin. Path.*, **55**, 283.


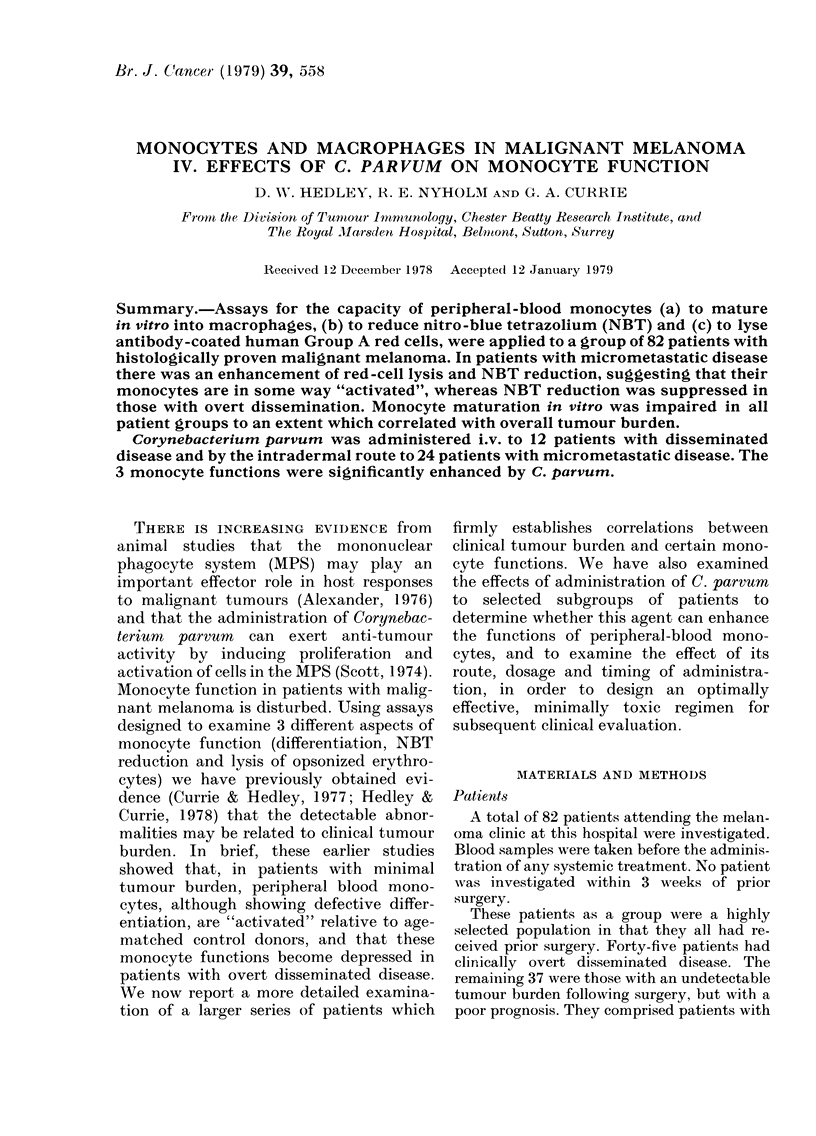

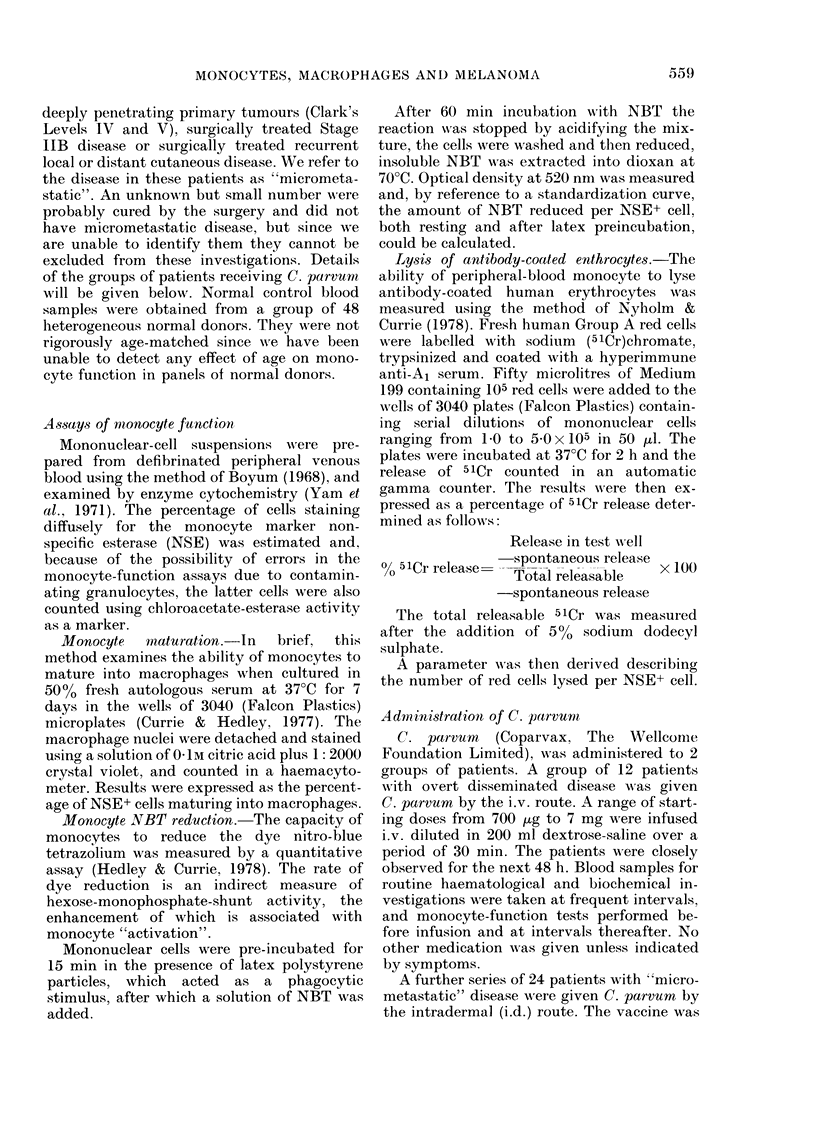

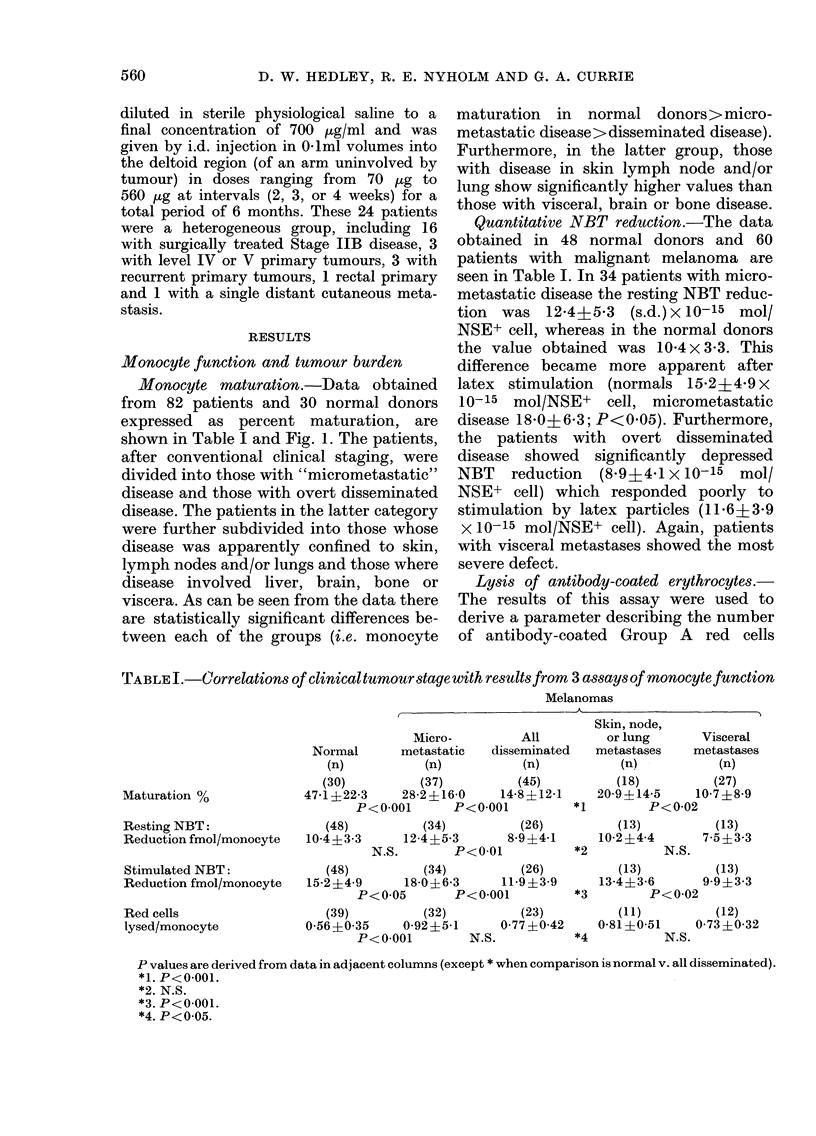

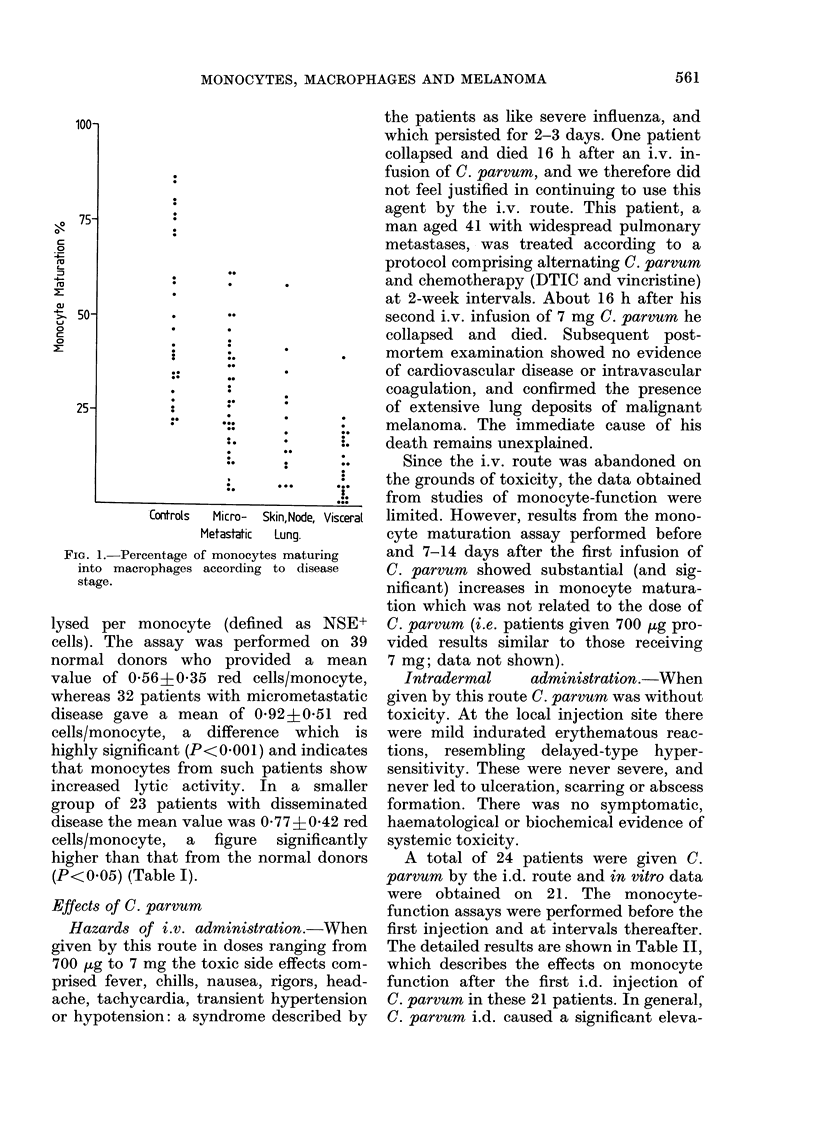

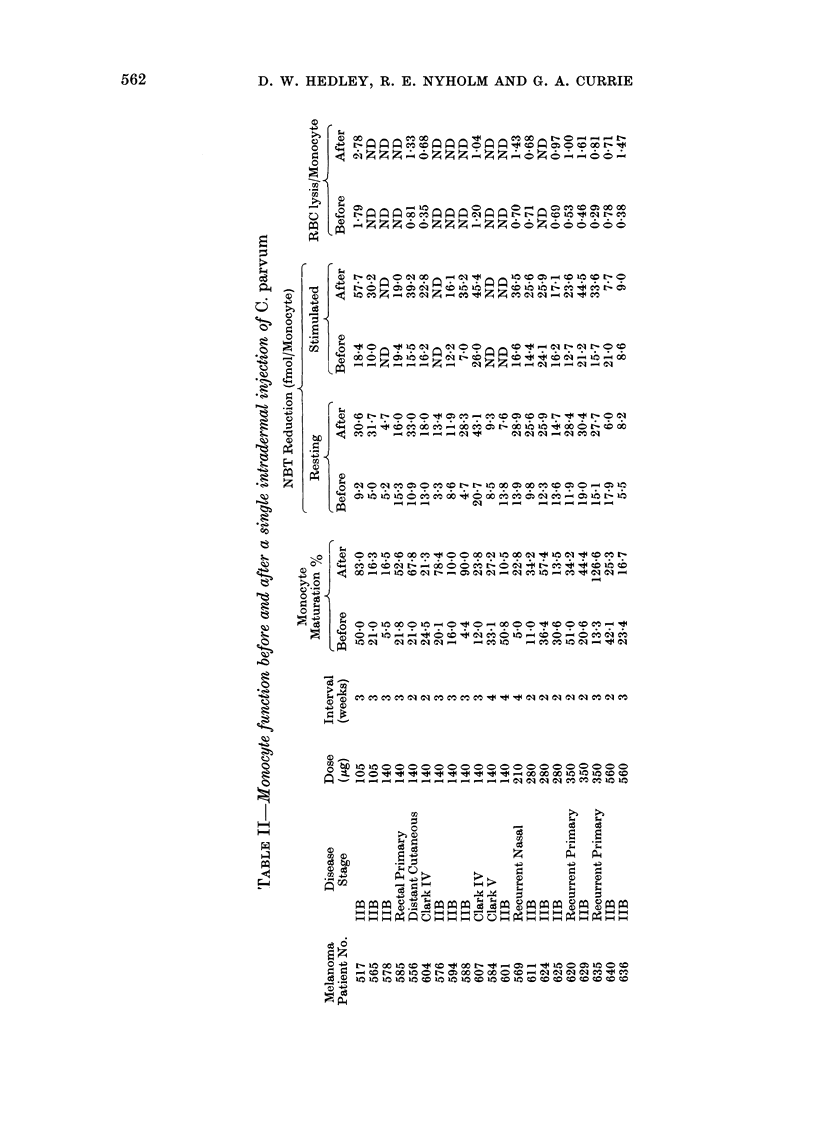

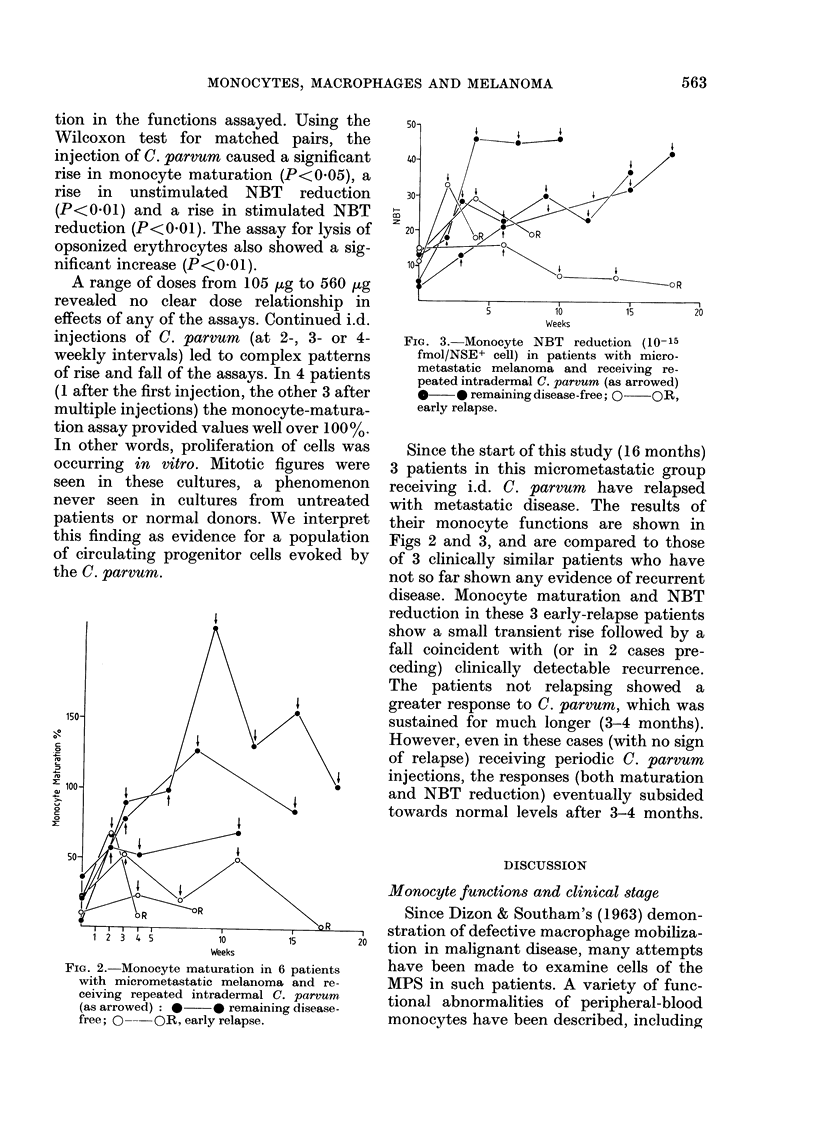

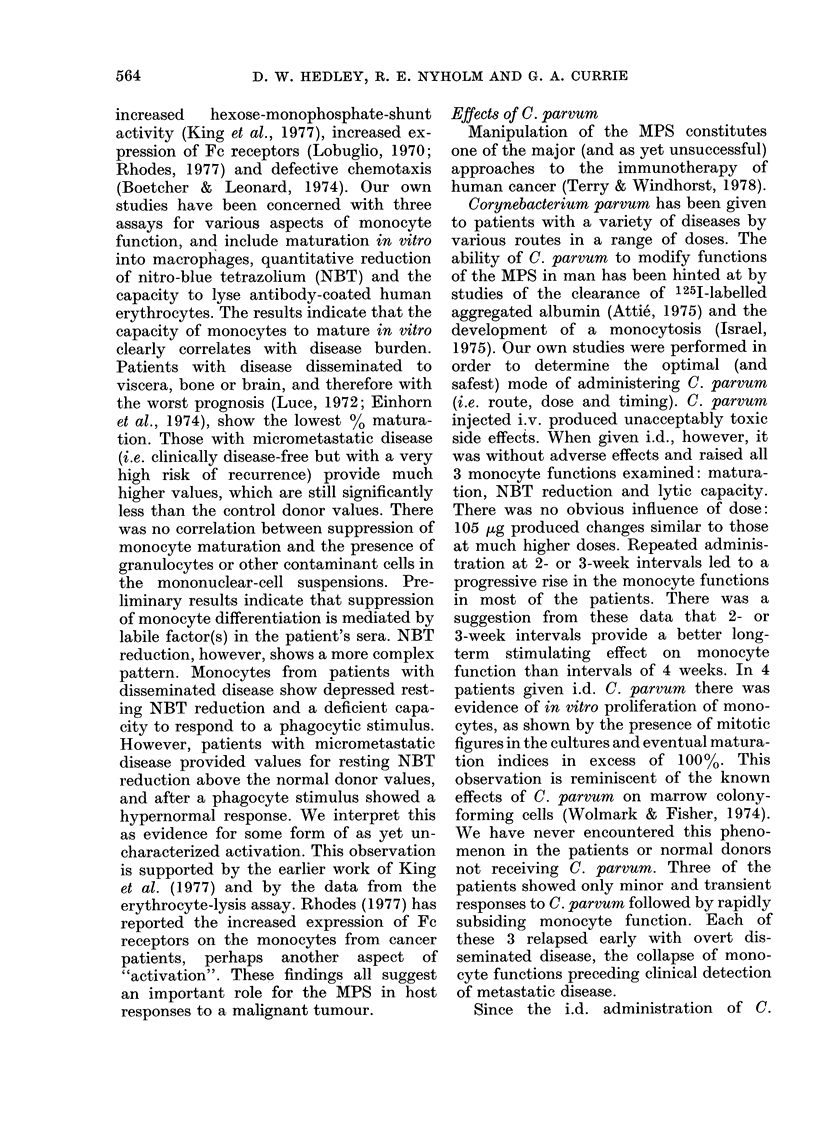

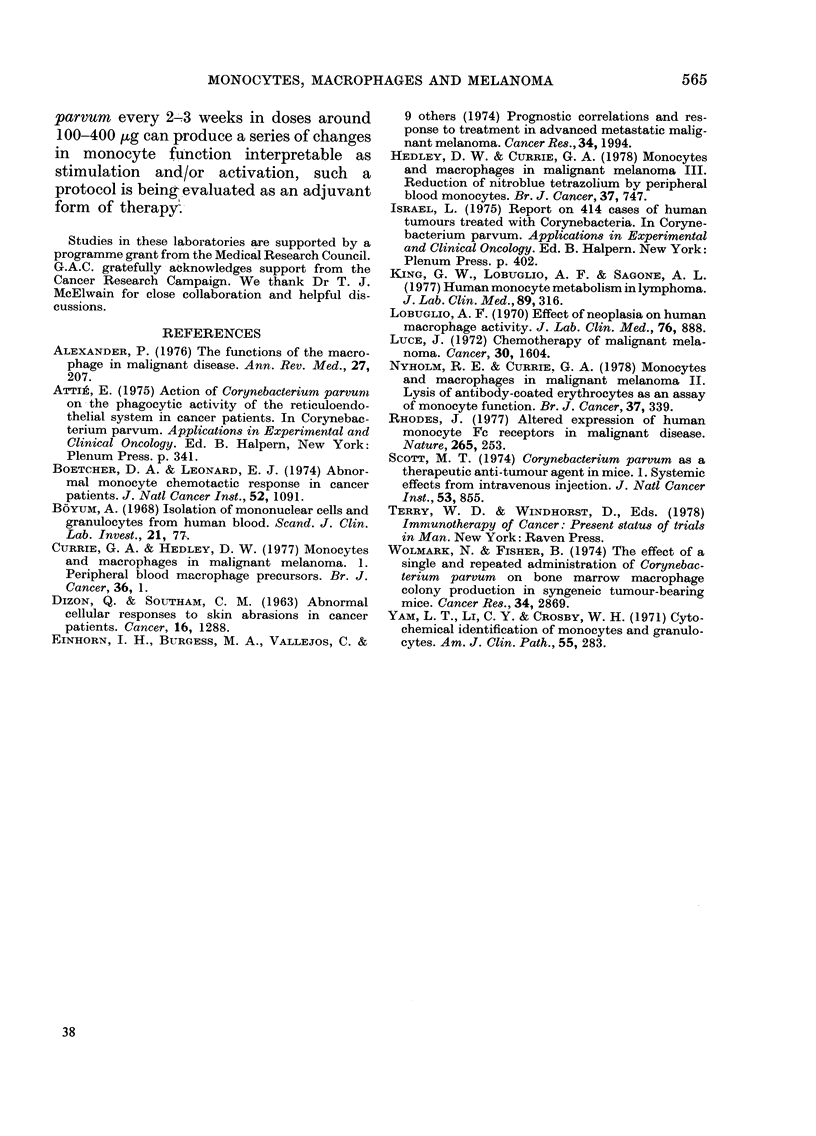


## References

[OCR_00829] Alexander P. (1976). The functions of the macrophage in malignant disease.. Annu Rev Med.

[OCR_00842] Boetcher D. A., Leonard E. J. (1974). Abnormal monocyte chemotactic response in cancer patients.. J Natl Cancer Inst.

[OCR_00847] Böyum A. (1968). Isolation of mononuclear cells and granulocytes from human blood. Isolation of monuclear cells by one centrifugation, and of granulocytes by combining centrifugation and sedimentation at 1 g.. Scand J Clin Lab Invest Suppl.

[OCR_00852] Currie G. A., Hedley D. W. (1977). Monocytes and macrophages in malignant melanoma. I. Peripheral blood macrophage precursors.. Br J Cancer.

[OCR_00858] DIZON Q. S., SOUTHAM C. M. (1963). ABNORMAL CELLULAR RESPONSE TO SKIN ABRASION IN CANCER PATIENTS.. Cancer.

[OCR_00871] Hedley D. W., Currie G. A. (1978). Monocytes and macrophages in malignant melanoma. III. Reduction of nitroblue tetrazolium by peripheral blood monocytes.. Br J Cancer.

[OCR_00884] King G. W., Lobuglio A. F., Sagone A. L. (1977). Human monocyte glucose metabolism in lymphoma.. J Lab Clin Med.

[OCR_00892] Luce J. K. (1972). Chemotherapy of malignant melanoma.. Cancer.

[OCR_00902] Rhodes J. (1977). Altered expression of human monocyte Fc receptors in malignant disease.. Nature.

[OCR_00907] Scott M. T. (1974). Corynebacterium parvum as a therapeutic antitumor agent in mice. I. Systemic effects from intravenous injection.. J Natl Cancer Inst.

[OCR_00918] Wolmark N., Fisher B. (1974). The effect of a single and repeated administration of Corynebacterium parvum on bone marrow macrophage colony production in syngeneic tumor-bearing mice.. Cancer Res.

[OCR_00925] Yam L. T., Li C. Y., Crosby W. H. (1971). Cytochemical identification of monocytes and granulocytes.. Am J Clin Pathol.

